# Emodin inhibits breast tumorigenesis in the comorbidity of hyperlipidemia and associated with IL-17 suppression

**DOI:** 10.1016/j.bbrep.2026.102520

**Published:** 2026-02-26

**Authors:** Qingqing Liu, Lujing Zheng, Chuangpeng Li, Peizhong Liu, Yu Ding, Qing Liu

**Affiliations:** a State Key Laboratory of Traditional Chinese Medicine Syndrome, Guangdong Provincial Hospital of Traditional Chinese Medicine, The Second Affiliated Hospital of Guangzhou University of Traditional Chinese Medicine, Guangzhou, 510120, PR China; b Department of Laboratory Animals, College of Animal Sciences, Jilin University, Changchun, Jilin, 130062, PR China; c Hebei Provincial Hospital of Traditional Chinese Medicine, Hebei Provincial Institute of Traditional Chinese Medicine Preparation Industry Technology, Shijiazhuang, 065900, PR China; d Guangdong Provincial Hospital of Traditional Chinese Medicine-Zhuhai Hospital, Zhuhai, 519015, PR China

**Keywords:** Breast cancer, Hyperlipidemia, Emodin, IL-17, Macrophages

## Abstract

**Background:**

This study explored the interaction between breast cancer and the hyperlipidemia microenvironment, and assessed the anti-tumorigenic effects of the natural compound Emodin.

**Methods:**

The human cancer atlas and gene expression databases were used to identify links between breast cancer and hyperlipidemia. Oxidized LDL (oxLDL) stimulation *in vitro* and high-fat diet (HFD) feeding *in vivo* were used to simulate hyperlipidemia. Quantitative PCR, flow cytometry, IF/IHC staining, FPLC and biological experiments were conducted to evaluate Emodin's efficacy. Molecular docking simulation and molecular dynamic analysis were used to identify potential targets of Emodin.

**Results:**

Lipid metabolism mediators CD36 and IL-17 activation were associated with breast cancer development. Bioinformatics identified IL-17 priming cytokines, and *in vitro* experiments confirmed Emodin inhibited Th17-priming cytokines after oxLDL stimulation. Emodin modulated tumorigenic genes especially apoptosis, inhibited breast cancer cell stemness and migration, and reduced tumor growth in HFD-feeding wild-type (WT) mice. Emodin reduced macrophage infiltration, angiogenesis, and IL-17 expression in tumors. Molecular docking and dynamic analysis suggested potential targets (IL17RA and TNFR1) for Emodin in modulating breast cancer development in hyperlipidemia microenvironment.

**Conclusion:**

Emodin effectively reduced tumorigenesis in HFD mice, accompanied with inhibited IL-17 expression and suppressed macrophage infiltration. This result provided evidence for the pro-tumorigenic role of hyperlipidemia in breast cancer development, and support the natural compound Emodin as a promising anti-tumor agent with targeting IL-17 signaling molecules.

## Highlights

• Bioinformatics and experimental evidence demonstrate that hyperlipidemia-related IL-17 signaling activation promote breast cancer progression.
• Emodin inhibits breast cancer cell stemness, migration, and *in vivo* tumor growth under hyperlipidemic conditions by suppressing IL-17 signaling and macrophage infiltration.
• Molecular docking identifies IL17RA and TNFR1 as potential targets of Emodin, supporting its candidacy as a natural anti-tumor agent targeting the hyperlipidemia-breast cancer crosstalk.

## 1 Introduction

Breast cancer patients frequently exhibit comorbidities with hyperlipidemia, and accumulating clinical evidence indicates that concurrent hyperlipidemia correlates with poorer prognosis in this population, including reduced disease-free survival and increased metastasis rates [1]. Emerging studies have begun to unravel the pathophysiological links between dysregulated lipid metabolism and breast cancer progression. Of particular interest is the oxidized low-density lipoprotein (oxLDL)-mediated immunomodulation within the tumor microenvironment. Experimental data reveal that oxLDL engages with the lectin-like oxidized LDL receptor-1 (LOX-1) on dendritic cells (DCs), triggering their maturation through upregulated expression of MHC class II molecules and co-stimulatory markers CD40/CD80/CD83/CD86 [2]. These activated DCs subsequently prime naive T cells to differentiate into interleukin-17 (IL-17)-producing T helper 17 (Th17) cells via IL-6/STAT3 signaling and TGF-β-dependent pathways.

The Th17-derived IL-17 establishes a pro-tumorigenic niche through multifaceted mechanisms: (1) Direct stimulation of cancer cell proliferation via STAT3-mediated upregulation of cyclin D1 [3], (2) Induction of angiogenic factors including VEGF and MMP9 to promote vascularization [4], and (3) Recruitment of myeloid-derived suppressor cells (MDSC) and pro-tumorigenic macrophages that impair antitumor immunity [4]. Notably, hyperlipidemia-induced oxLDL accumulation creates a feed-forward loop by enhancing DC-mediated Th17 polarization while simultaneously inducing lipid peroxidation in cancer cells, thereby promoting epithelial-mesenchymal transition (EMT) and metastatic dissemination [5]. This lipid-inflammation axis provides novel insights into the metabolic regulation of tumor immunity and suggests potential therapeutic targets for breast cancer patients with hyperlipidemia comorbidity.

Natural compounds have emerged as promising therapeutic agents in oncology due to their multi-target efficacy and favorable safety profiles. Paclitaxel (PTX), a classical microtubule-stabilizing agent derived from Taxus species, has been widely utilized in breast cancer treatment by arresting mitotic progression through β-tubulin polymerization [6]. However, drug resistance and systemic toxicity necessitate the exploration of complementary phytochemicals. Another natural compound, Emodin (1,3,8-trihydroxy-6-methylanthraquinone), a bioactive anthraquinone isolated from *Rheum palmatum* and *Polygonum cuspidatum*, has garnered attention for its broad-spectrum antitumor activities. Our preclinical studies demonstrate that Emodin suppresses BC progression through multiple mechanisms including cell cycle arrest, apoptosis induction, anti-angiogenesis and metastasis suppression via modulation of EMT markers [7,8].

However, it is unknown whether Emodin could inhibit breast tumorigenesis in the comorbidity of hyperlipidemia. Thus, this study was designed to explore the role of Emodin in modifying breast tumorigenesis, especially in the oxidized lipid-activated IL-17 microenvironment in breast cancer.

## 2 Methods

### 2.1 Database analysis

The database of human cancer altas (HPCA) were used to analyze the expression of CD36, IL17A and IL17RA in breast cancer samples. The gene expression databases (GeneCards, OMIM and NCBI genes) were used to explore the interaction between diseases of breast cancer, hyperlipidemia and IL-17 signaling pathways, by searching the keywords of these two diseases. The TCMSP and TTD database were used to explore the targets of Emodin, which was used for the construction of compound-targets network. The STRING database was used to construct the generic PPI network based on the results from Venn intersection. The hub gene screening and functional enrichment of Gene Ontology (GO) and KEGG analysis were conducted based on the compound-targets network.

### 2.2 Cell culture and compounds treatment

DC2.4 cells (iCell-m016) were cultured in RPMI 1640 medium containing 2 mM l-glutamine, 100 μg/ml streptomycin, 100 U/ml penicillin, 100 μM non-essential amino acids, 50 μM 2-mercaptoethanol, and 10% fetal bovine serum (FBS). E0771 cells (iCell-m090) were maintained in high-glucose DMEM (HEPES) supplemented with 10% FBS and 1% penicillin/streptomycin (PS). All cell lines were incubated at 37 °C in 5% CO_2_. For induction, DC2.4 cells were pretreated with serum-free medium (SFM) and then treated with 25 μM Emodin and 50 μg/ml oxidized low-density lipoprotein (ox-LDL) for 24 h. Similarly, E0771 cells were pretreated with SFM and subsequently treated with 25 μM Emodin and 20 ng/ml mouse IL-17A protein for 24 h.

### 2.3 Animal ethics, high-fat diet (HFD) feeding and compounds treatment

The female C57BL/6J mice were purchased from Guangdong Yaokang Biotechnology Co., LTD. The positive drug 20 mg/kg Paclitaxel (PTX, HY-B0015) and the therapeutic drug 40 mg/kg Emodin (HY-14393) were administered intraperitoneally (i.p.) once every 2 days for a total of 7 days. The mice in each group were fed ordinary diet or western diet (Fat 21%, cholesterol 0.15%, protein 15.5%; Guangdong Medical Laboratory Animal Center) for 17 ∼ 32 days. All experiments were performed at the Experimental Animal Ethics Committee of Guangdong Hospital of Traditional Chinese Medicine under an approved project license (No. 2024050).

### 2.4 Quantitative PCR

Total RNA was isolated from cells using an RNA extraction kit (Sevenbio, Beijing, China). cDNA was synthesized from the RNA using the Color All-in-one Reverse Transcription Kit (with DNase; EZBioscience, USA). Real-time quantitative PCR was performed using the ViiA™7 system (Applied Biosystems, USA) with 2x Color SYBR Green gPCR Master Mix (ROX2 plus; EZBioscience). The thermal cycling conditions were as follows: initial denaturation at 95 °C for 5 min, followed by 40 cycles of 95 °C for 10 s and 60 °C for 30 s, with a final hold at 4 °C. The expression levels of target genes were quantified using the 2–ΔΔCt method. Primer sequences are listed in Table 1.

**Table 1:** Mouse Primers used for qRT-PCR.

Target gene	Sequence (5′to 3′)	
IL-6	Forward primer	CTTCCATCCAGTTGCCTTCT
	Reverse primer	CTCCGACTTGTGAAGTGGTATAG
IL-1β	Forward primer	GGTGTGTGACGTTCCCATTA
	Reverse primer	ATTGAGGTGGAGAGCTTTCAG
TNF-α	Forward primer	TTGTCTACTCCCAGGTTCTCT
	Reverse primer	GAGGTTGACTTTCTCCTGGTATG
TGF-β	Forward primer	CCGCAACAACGCCATCTATG
	Reverse primer	GGATCCACTTCCAACCCAGG
CD36	Forward primer	CAGGAGTGCTGGATTAGTGGTT
	Reverse primer	CATGCAGTGCAGAAGGGTG
Caspase-3	Forward primer	CAGTGGACTCTGGGATCTATCTG
	Reverse primer	TGACATTCCAGTGCTCTTATGG
Caspase-9	Forward primer	CAGAGGCTGTTAAACCCCTAGAC
	Reverse primer	TCCAGCTTCACTACTCTCTGCTC
TP-53	Forward primer	ACTCTCCTCCCCTCAATAAGCTA
	Reverse primer	GTGCTGTGACTTCTTGTAGATGG
Bax	Forward primer	ATATGGAGCTGCAGAGGATGAT
	Reverse primer	CAAAGTAGAAGAGGGCAACCAC
Bcl2	Forward primer	GTGGATGACTGAGTACCTGAACC
	Reverse primer	AGCCAGGAGAAATCAAACAGAG
N-cad	Forward primer	GGGACAGGAACACTGCAAAT
	Reverse primer	CGGTTGATGGTCCAGTTTCT
MMP2	Forward primer	CAAGTTCCCCGGCGATGTC
	Reverse primer	TTCTGGTCAAGGTCACCTGTC
Vim	Forward primer	ATGCTTCTCTGGCACGTCTT
	Reverse primer	AGCCACGCTTTCATACTGCT
LOX1	Forward primer	AGGTCCTTGTCCACAAGACTGG
	Reverse primer	ACGCCCCTGGTCTTAAAGAATTG
CCL2	Forward primer	GTGCTGACCCCAAGAAGGA
	Reverse primer	TGAGGTGGTTGTGGAAAAGG
IL-17A	Forward primer	TTTAACTCCCTTGGCGCAAAA
	Reverse primer	CTTTCCCTCCGCATTGACAC
IL-23	Forward primer	ATCTTCAAAGGGGAGCCTGC
	Reverse primer	GCTGCCACTGCTGACTAGAA
Tubulin	Forward primer	AGCAGCTACTTTGTGGAGTG
	Reverse primer	TCGGAGATGCGCTTGAATAG

### 2.5 Serum cholesterol examination by lipid kit and Fast Protein Liquid Chromatography (FPLC) analysis

For serum lipid profile (VLDL, LDL, and HDL cholesterol) detection, mouse peripheral blood samples from control diet (CD) and high-fat diet (HFD) groups were first collected via orbital venous plexus, centrifuged at 3000 rpm for 10 min at 4 °C to separate serum. For serum total cholesterol, the mouse total cholesterol ELISA kit (ab285242) was used for detection. For cholesterol components examination, 200 μL of serum was loaded onto a size-exclusion chromatography column connected to a Fast Protein Liquid Chromatography system, eluted with phosphate-buffered saline (pH 7.4) at a flow rate of 0.5 mL/min, and 0.5 mL fractions were collected sequentially. The cholesterol concentration in each fraction was quantified using an enzymatic colorimetric cholesterol assay kit, and cholesterol levels were plotted against fraction numbers to distinguish VLDL, LDL, and HDL fractions.

### 2.6 Flow cytometry

First, lymph nodes and peripheral blood cells were isolated from experimental mice. The cell suspension was collected in centrifuge tubes, and red blood cells were lysed using red blood cell lysis buffer. The cells were washed with PBS, and the supernatant was removed by centrifugation. The cell concentration was adjusted to 1 × 10^6^ cells/ml. Subsequently, an appropriate amount of FC Block was added to the cells, which were incubated at 4 °C in the dark for 15 min to block nonspecific binding sites. Afterward, the supernatant was discarded, and a pre-diluted primary antibody cocktail was added to the cells, which were then incubated at 4 °C in the dark for 30 min. After incubation, the cells were washed 2-3 times with buffer (PBS containing 2% FBS), and the supernatant was removed by centrifugation each time. The cells were then resuspended in buffer. Finally, the cells were resuspended in an appropriate volume of buffer and prepared for flow cytometry analysis. Throughout the process, it is important to maintain the cells at low temperature and to thoroughly remove the supernatant after each wash to avoid residual antibodies that could affect the subsequent detection results. Antibodies from the following suppliers were used: ThermoFisher Scientific: FC Block (14-0161-81), CD45 (11-0451-82), CD4 (48-0041-82), CD11B (12-0112-82); CD11C (558079, BD Biosciences)

### 2.7 IHC staining

Breast cancer tissue sections were mounted onto glass slides and fixed with 4% paraformaldehyde for 15 ∼ 30 min. Antigen retrieval was performed by heating the sections in citrate buffer (pH 6.0) using a microwave. After cooling, the sections were washed with PBS, followed by the addition of a 3% hydrogen peroxide solution and incubation at room temperature for 10 min to quench endogenous peroxidase activity. The sections were then washed with PBS and blocked with a blocking solution (5% BSA or normal goat serum) at room temperature for 30 min to minimize nonspecific binding. Primary antibodies against CD68 (GB113109), VEGFA (GB15165), and IL-17RA (bs-2606R) were applied to the sections and incubated overnight at 4 °C. The following day, the sections were washed with PBS and incubated with secondary antibodies (typically horseradish peroxidase-conjugated or fluorescently labeled) at room temperature for 30 min. Finally, the sections were washed with PBS, and the chromogenic substrate DAB was added for color development. The sections were then observed and photographed under a microscope.

### 2.8 Wound-healing assay

E0771 cells were cultured in 6-well plates until they reached near 100% confluence. The 200 μL pipette tips were used to create linear scratches across the monolayer, ensuring unifoerilrmity and consistency. The cells were gently washed twice with PBS to remove debris generated from the scratching process. Basal medium supplemented with various drugs was then added to promote cell migration. Images of the initial scratch were captured at 0 h, followed by images taken at the same location at the predetermined time point (24 h) to assess cell migration. The migration capacity of the cells was evaluated by measuring the changes in scratch width.

### 2.9 Molecular docking simulation by AutoDock

The database of the Research Collaboratory for Structural Bioinformatics PDB (RCSR PDB) was used to check the target protein structures, and the NCBI PubChem database was used to find the docking ligand of Emodin. After obtaining the molecular structures of the target proteins and ligand compounds, the PyMOL software was used to remove H_2_O and ligands. Then the molecular docking stimulation of the target proteins and ligand compounds was conducted in the software of AutoDock. The binding energy of less than −5.0 kcal/mol was characterized as good binding activity, and the lower binding energy indicated the greater probability of binding activity.

### 2.10 Molecular dynamic analysis

To validate the molecular docking results, molecular dynamic analysis were performed based on the docking results. Briefly, the protein-ligand complexes were conducted using the GROMACS 2025.3 software package [9]. The system was placed in a periodic boundary cubic box and solvated using the AMBER14SB force field [10] and the TIP3P water model. Small molecule topology files were generated with the Sobtop software, and ions were added to achieve physiological concentrations (150 mM NaCl). Energy minimization was performed for 500 steps using the conjugate gradient algorithm, followed by 100 ps (time step 1 fs) restrained dynamics simulations and 100 ns (time step 2 fs) production simulations. Temperature was maintained at 298.15 K via the V-rescale method (time constant 0.2 ps), and pressure was kept at 1.0 bar using the C-rescale method (time constant 0.5 ps/2.0 ps). LINCS was employed to constrain hydrogen bonds, long-range electrostatics were handled by PME (cutoff radius 1.0 nm), van der Waals interactions were treated with Verlet cutoff (cutoff radius 1.0 nm), and energy and pressure dispersion corrections were enabled. During the simulation, the center of mass motion of the system was removed, and in the production phase, the protein-ligand complex was subjected to angular momentum removal and independent temperature coupling.

### 2.11 Statistical analysis

The GraphPad Prism 9 software (GraphPad Software, SanDiego, CA) was used to carry out all statistical analysis. Student's *t*-test analysis were employed for comparisons between two groups. One-way ANOVA analysis of variance was used for multiple groups comparisons. If the data followed a Gaussian distribution, then Bonferroni's multiple comparisons were used as a posttest. Otherwise, the nonparametric Kruskal-Wallis test and Dann's multiple comparison posttest were used to analyze the data. A P value of <0.05 was considered as statistically significant.

## 3 Results

### 3.1 The lipid metabolism mediators were associated with breast cancer development in human breast cancer database

**Fig. 1:**
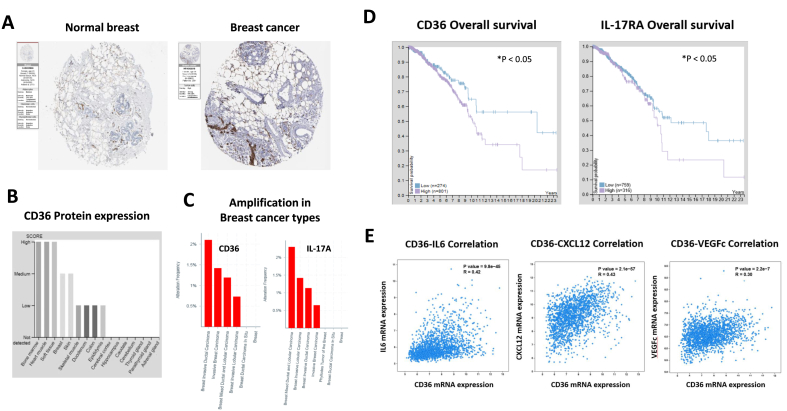
**The lipid metabolism mediator CD36 and IL17 expression levels were associated with breast cancer development.** The pathological tissue slice from HPCA database showed different characters in normal breast versus breast cancer (A.). CD36 expression showed high levels in bone marrow and heart among different tissues (B.). The amplification of CD36 and IL-17 showed highest levels in more invasive breast cancer types than the normal types (C.). The CD36 and IL-17 expression levels were correlated with overall survival (D.) of breast cancer and tumorigenic markers including IL-6, CXCL12 and VEGF (E.).

Among the screening of lipid metabolism mediators in HPCA database, the lipid uptake receptor CD36 was found to be upregulated in the breast cancer biopsy tissue. CD36 exhibited higher level in malignant breast cancer than benign tumor (Fig. 1A–C). As a classical ligand of CD36, the oxidized LDL (oxLDL) activates dendritic cells (DC) and primes Th17 cells to produce IL-17. Survival analysis reveled that CD36 and IL17RA were negatively correlated with overall survival of breast cancer patients, and positively correlated with tumorigenic proteins including IL-6, CXCL12 and VEGF (R > 0.3, P < 0.05) (Fig. 1D and E).

### 3.2 The IL-17 priming pathway was involved in breast cancer and hyperlipidemia, indicated by bioinformatics and oxLDL-primed dendritic cells (DC)

**Fig. 2:**
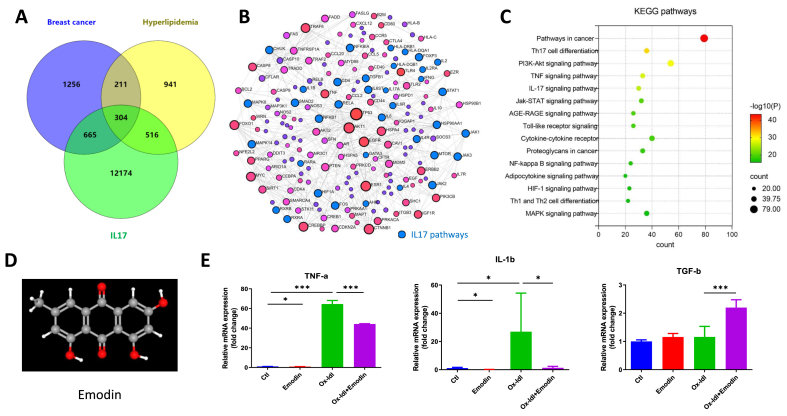
**The IL-17 priming pathway was involved in breast cancer and hyperlipidemia.** The bioinformatics exploration with GeneCards, OMIM and NCBI genes databases were used to present the interaction between diseases of breast cancer, hyperlipidemia and IL-17 signaling pathways (A.). The constructed gene network using overlapped genes from (A.) showed high proportion of the participation of IL-17 pathways (labeled blue) (B.). The bubble plot showed KEGG functional pathways in the comorbidity of breast cancer and hyperlipidemia (C.). Emodin (D.) inhibited the key cytokines for oxLDL-primed IL-17 activation in DC2.4 cell line by PCR test (E.). ∗P < 0.05, ∗∗∗P < 0.001.

The bioinformatics exploration showed intersection (304 common genes) among the gene subsets with breast cancer, hyperlipidemia and IL-17 signaling activation. Generic protein-protein interaction (PPI) network and KEGG functional enrichment indicated IL-17 was associated with the closed relationship between hyperlipidemia and breast cancer development (Fig. 2A–C). To check whether Emodin could inhibit cytokines production by DC for Th17 priming, emodin was used as a natural compound for drug intervention. Results showed Emodin reversed the key cytokines (TNF-a, IL-1b and TGF-b) production from DC2.4 cell line, which were important mediators for oxLDL-primed Th17 activation (Fig. 2D and E).

### 3.3 Emodin modulated tumorigenic genes in breast cancer cells in the absence or presence of IL-17 stimulation

**Fig. 3:**
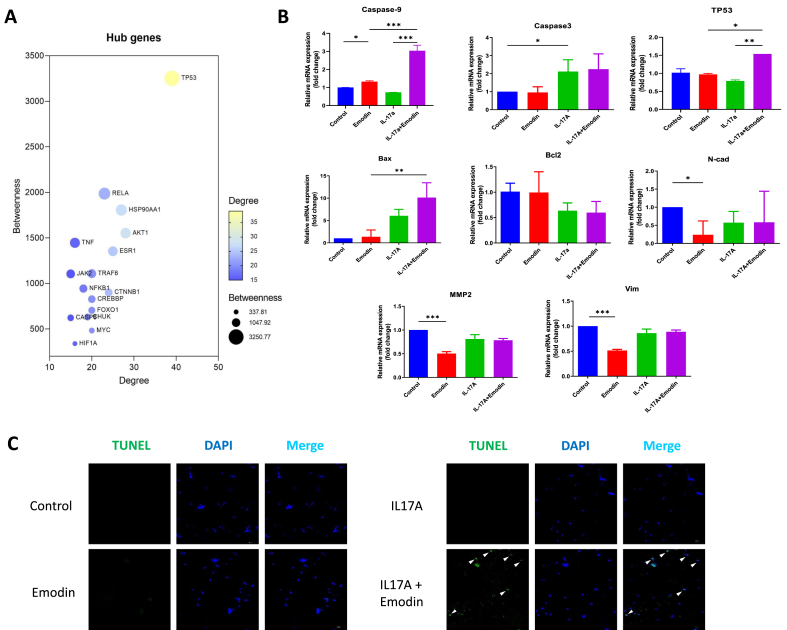
**Emodin modulated tumorigenic genes in breast cancer cells in the absence or presence of IL-17 stimulation.** The hub tumorigenic genes from Fig. 2B were quantified by betweeness and degree (A.). The tumorigenic genes were tested in E0771 breast cancer cells in the absence or presence of IL-17A stimulation (B.). The TUNEL staining of E0771 breast cancer cells in the absence or presence of IL-17A and/or Emodin stimulation (C.). ∗P < 0.05, ∗∗P < 0.01, ∗∗∗P < 0.001.

Regarding to the breast cancer cell *per se*, the hub tumorigenic genes in database were screened. Both the hub genes and classically recognized genes for tumorigenesis were tested in E0771 breast cancer cells (Fig. 3A). Quantitative PCR result showed the suppressive effect of Emodin for tumorigenesis, including the upregulation of breast cancer cell death (caspase-9, caspase-3, bax), inducing antitumor gene (TP53), and downregulating epithelial-mesenchymal transition genes (N-cadherin, vimentin), tumor metastasis gene (MMP2). Notably, Emodin exerted significant anti-tumorigenic effect in modulating caspase-9, TP53 and bax in the presence of IL-17 stimulation (Fig. 3B). To validate the pro-apoptotic effect of Emodin, TUNEL staining was performed, and results were consistent to PCR that Emodin treatment increased apoptosis of E0771 breast cancer cells in the IL-17 stimulation microenvironment (Fig. 3C).

### 3.4 Emodin inhibited the stemness and migration properties of breast cancer cells in the absence or presence of IL-17 stimulation

**Fig. 4:**
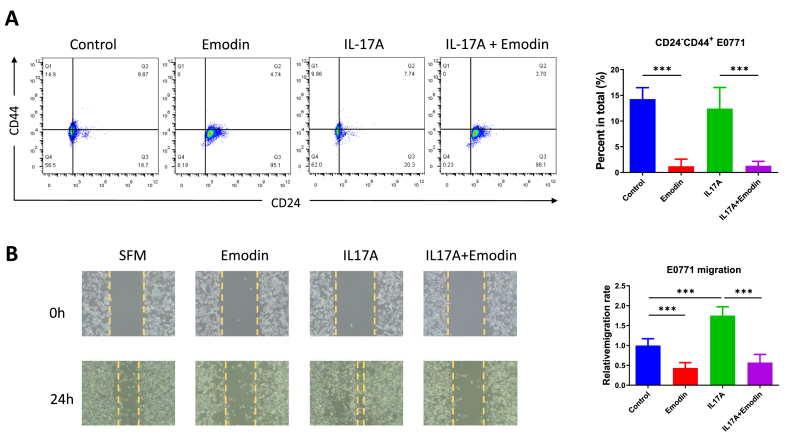
**The tumorigenic stemness and migration properties of breast cancer cells were examined by Emodin in the absence or presence of IL-17 stimulation.** The cell stemness of E0771 breast cancer cells were examined by flow cytometry after the treatment of Emodin in the absence or presence of IL-17 stimulation (A.). The cell migration of E0771 breast cancer cells were examined by wound healing assay after the treatment of Emodin in the absence or presence of IL-17 stimulation (B.). ∗∗∗P < 0.001.

Based on the modulated tumorigenic genes by Emodin in Fig. 3, we further verified in their biology effects. The flow cytometry analysis by staining CD24^−^CD44^+^ population in E0771 breast cancer cells suggested the proportion of progenitor and stem cells were reduced by Emodin treatment, even in the IL-17A stimulated micro-environment (Fig. 4A). The scratch experiment in E0771 cells evidenced that IL-17A increased breast cancer migration ability, while this property was inhibited by Emodin both in the absence and presence of IL-17 stimulation (Fig. 4B).

### 3.5 High fat diet (HFD)-feeding increased breast cancer development, accompanied by elevated cholesterol and pro-tumorigenic macrophages

**Fig. 5:**
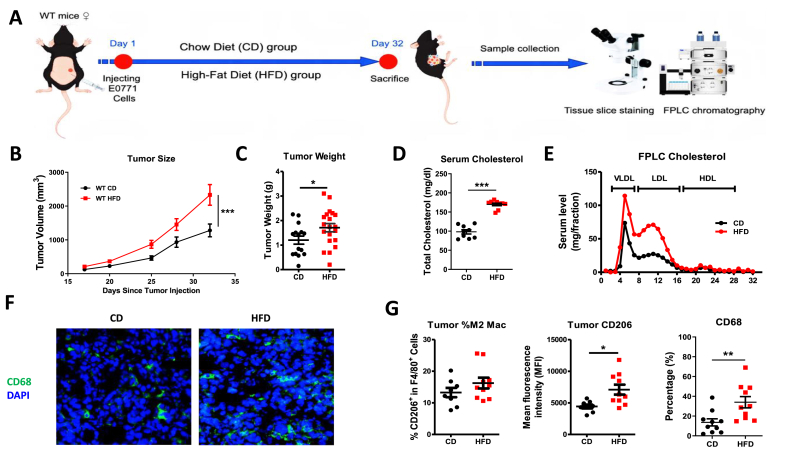
**High fat diet (HFD) increased breast cancer growth and elevated serum lipid levels in E0771 tumor-bearing mice.** Tumor volume (A.) and tumor weight (B.) were measured after E0771 cell implantation with chow diet or high fat diet feeding. Serum lipids were tested by total cholesterol (C.) and FPLC analysis (D.). Immunofluorescence staining (F.) and flow cytometry analysis (G.) showed increased macrophages in tumor tissue. ∗P < 0.05, ∗∗P < 0.01, ∗∗∗P < 0.001.

To explore the pro-tumorigenic effect of hyperlipidemia, the HFD-feeding mice were used to mimic hyperlipidemia microenvironment in breast cancer development. The WT mice were fed by HFD for 32 days, and breast cancer cells were implanted into both sides of mammary glands. The tumor growth and tumor weight were both higher than that in the HFD group compared with chow diet group (P < 0.05) (Fig. 5A–C). Lipid examination by kit and FPLC showed serum cholesterol especially LDL cholesterol was more significant in the HFD group (P < 0.05) (Fig. 5D and E). IF staining of the tumor slice and flow cytometry demonstrated that the proportion of pro-tumorigenic macrophages (M2-like phenotype) was significantly increased in the HFD group (P < 0.05), which suggested these macrophages played important roles in breast cancer development (Fig. 5F and G).

### 3.6 Emodin reduced breast tumor growth in the HFD-feeding mice, accompanied by suppressing pro-tumorigenic macrophages and IL-17 signaling

**Fig. 6:**
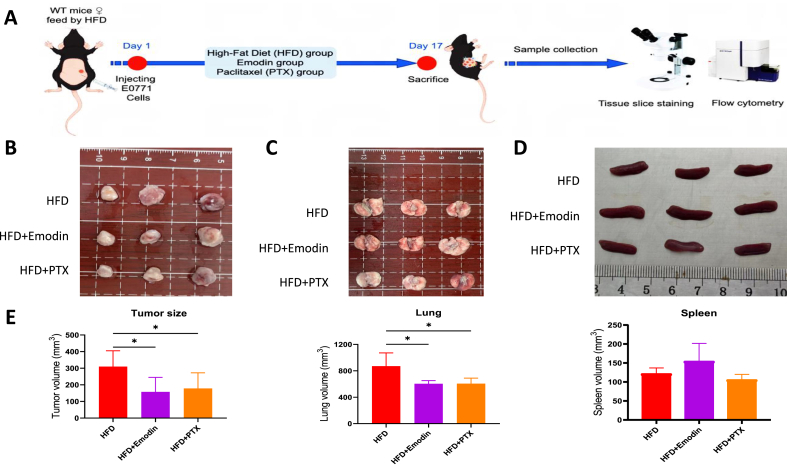
**Emodin reduced breast tumor growth in the high fat diet (HFD)-feeding mice.** The workflow diagram for animal experiment (A.). The breast tumor volume (B/E.) were checked in Emodin treatment or paclitaxel (PTX). The morphology of lung (C/E.) and spleen (D/E.) were tested in Emodin treatment or PTX. ∗P < 0.05.

Then the anti-tumorigenic effect of Emodin was tested in the HFD-feeding mice, and compared with paclitaxel (PTX), which was used as a positive control. Compared with the HFD model group, Emodin or PTX treatment markedly reduced breast cancer growth (P < 0.05) (Fig. 6A and B). There was no significant metastasis shown in the lung surface, however, the lung volume in HFD group treatment with Emodin was less than that in the HFD group (P < 0.05) (Fig. 6C). The spleen volume showed a increased trend after Emodin treatment in the HFD-feeding mice (Fig. 6D).

### 3.7 Emodin inhibited macrophages recruitment, angiogenesis and IL-17 signaling in tumor, but not CD4^+^T cell proportion

**Fig. 7:**
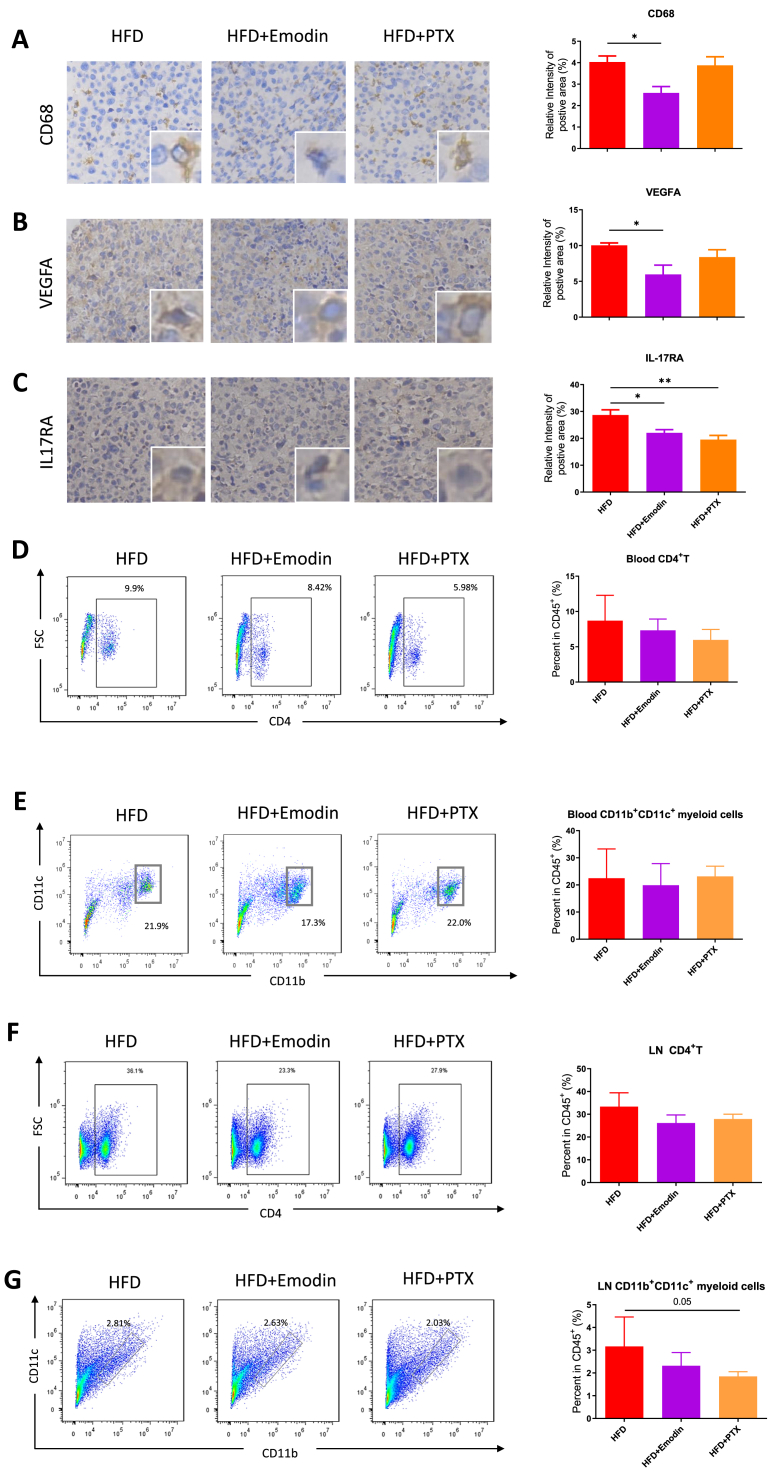
**Emodin inhibited macrophages infiltration, angiogenesis indicators and IL-17 receptor expression in breast tumor tissue.** The CD68 (A.), VEGFA (B.) and IL17RA (C.) were stained and examined by IHC in tumor tissue with Emodin treatment or paclitaxel (PTX). The cell proportions of CD4^+^T cells and dendritic cells (DC) that affected tumor growth were detected in peripheral blood (D-E.) and lymph nodes (F-G.) by flow cytometry. ∗P < 0.05, ∗∗P < 0.01.

The immunocytes infiltration and biological functions for tumorigenesis were checked by IHC and flow cytometry analysis in tumor tissue. The CD68^+^ macrophages in the Emodin treatment group were obviously decreased compared to the HFD group (P < 0.05) (Fig. 7A). The VEGFA^+^ angiogenesis marker was also significantly decreased in the Emodin treatment group, but not the HFD-PTX group (Fig. 7B). The IL17RA^+^ region in the Emodin or PTX treatment group was significantly decreased compared to the HFD group (P < 0.05) (Fig. 7C). Regarding to the other immunocytes, flow cytometry detection in peripheral blood showed that CD4^+^T cells in Emodin or PTX treatment group was not significantly changed compared to HFD group (P > 0.05). The proportion of blood CD11b^+^CD11c^+^ myeloid cells, normally consistent of dendritic cells and macrophages, was also not significantly changed after Emodin treatment (Fig. 7D and E). Moreover, the cell proportions in the draining lymph nodes were also detected by flow cytometry. Although there was no statistical significance, the changes in lymph nodes was consistent to that in the peripheral blood samples (Fig. 7F and G).

### 3.8 The tumorigenic genes and IL-17 activation-associated genes were regulated by Emodin in breast cancer tissue

**Fig. 8:**
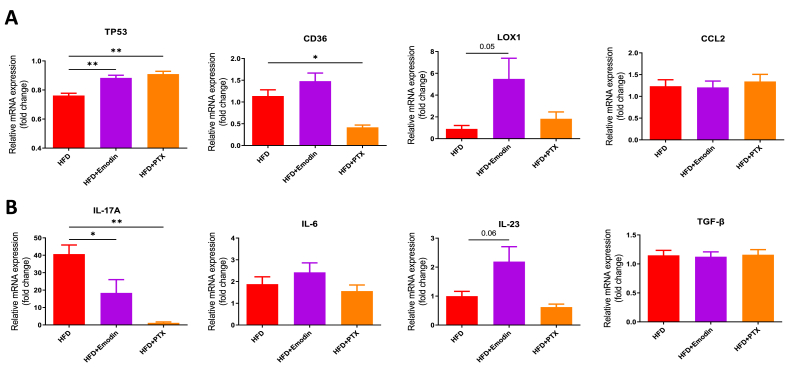
**The tumorigenic genes and IL-17 activation-associated genes were regulated by Emodin in breast cancer tissue.** RNA from breast cancer tissue were extracted and PCR were performed for gene analysis, including tumorigenic genes (A.) and IL-17 activation-associated genes (B.). ∗P < 0.05, ∗∗P < 0.01.

Furthermore, the transcription levels of the tumorigenic genes and IL-17 activation-associated genes were examined by quantitative PCR in tumor tissue. Results indicated that Emodin or PTX treatment significantly upregulated TP53 expression in the HFD-Emodin group (P < 0.05). The lipid metabolism receptors CD36 and Lox1 were also tested for oxLDL uptake in the tumor tissue, but not reached statistical significance (P > 0.05). Checking with the IL-17 activation-associated genes showed that the IL-17A was significantly downregulated by Emodin or PTX treatment (P < 0.05), but not the other related cytokines (P > 0.05) (Fig. 8).

### 3.9 Molecular docking simulation indicated the targets for Emodin in modulating breast cancer development in the comorbidity of hyperlipidemia

**Fig. 9:**
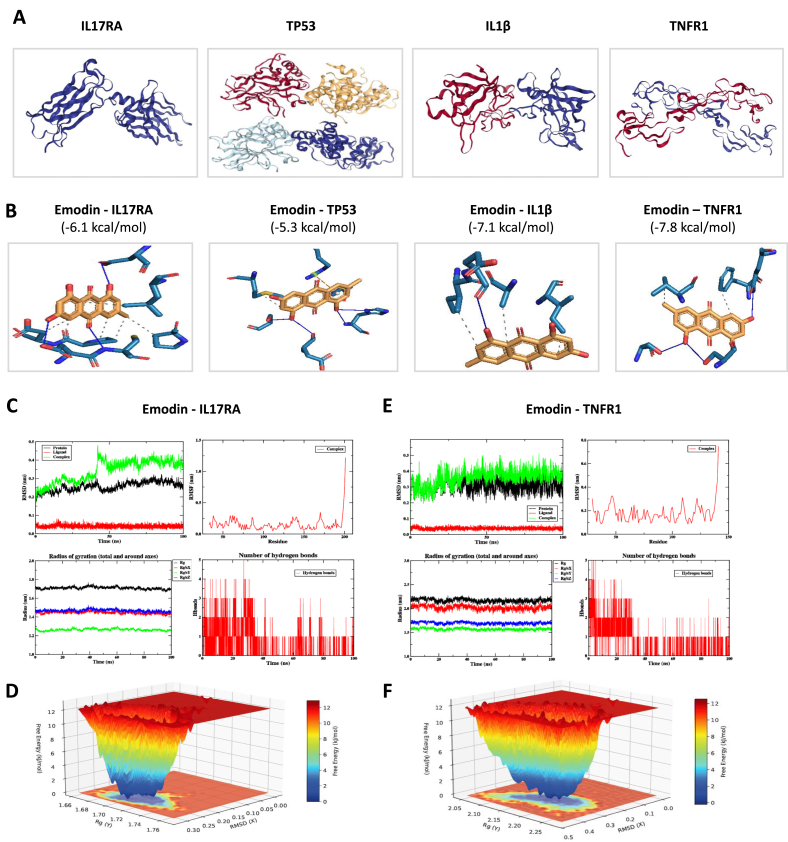
**Molecular docking simulation indicated the targets for Emodin in modulating breast cancer development in the comorbidity of hyperlipidemia.** The chemical structures of selected targets (A.) and potential binding position for Emodin (B.). The molecular dynamic analysis of the binding effects of Emodin and IL17RA/TNFR1(C–F.). The root mean square deviation (RMSD), root mean square fluctuation (RMSF), radius of gyration (Rg), H-bonds (H) were evaluated in the binding between Emodin and IL17RA/TNFR1(C.E.). The 3D free energy landscape (FEL) indicated the binding energy between Emodin and IL17RA/TNFR1 (D.F.).

Regarding to the targets for Emodin in modulating breast tumorigenesis, the candidate targets including IL17RA, TP53, IL1β and TNFR1 were used for molecular docking simulation. The chemical structures of selected targets were shown and potential binding position for Emodin were predicted. All of the binding energy for the targets to combine with Emodin was less than the normal threshold (−5.0 kcal/mol), indicating that these targets were potential targets for Emodin (Fig. 9).

To validate the results from molecular docking simulation, molecular dynamic analysis was performed, including root mean square deviation (RMSD), root mean square fluctuation (RMSF), radius of gyration (Rg), hydrogen (H)-bonds and free energy landscape analysis (FEL). For the Emodin-IL17RA complex, IL17RA backbone RMSD stabilized quickly after an initial transient increase with minimal fluctuations, and Emodin RMSD remained low, confirming stable binding. RMSF showed minor fluctuations in the flexible surface regions of IL17RA, while Rg values stayed constant. The Emodin-IL17RA interface maintained 1-3 stable hydrogen bonds, and FEL analysis identified a deep energy well at Rg ≈ 1.72 Å and pMSD ≈0.3 Å (Fig. 9C and D). Similar stability was observed for the Emodin-TNFR1 complex. TNFR1 backbone and Emodin RMSD values remained stable, and Rg values stayed constant. The interface maintained 1-3 stable H-bonds, and FEL analysis revealed a deep energy well at Rg ≈ 2.15 Å and pMSD ≈0.4 Å, confirming a thermodynamically dominant conformation (Fig. 9E and F).

**Fig. 10:**
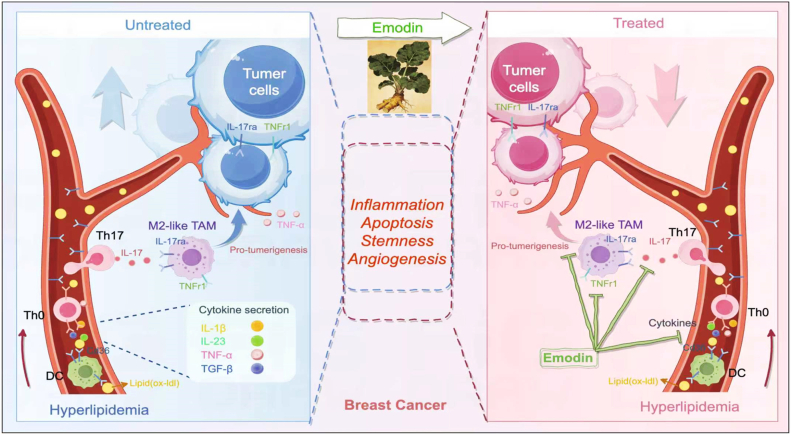
**The schematic diagram for Emodin in modulating breast cancer development in the comorbidity of hyperlipidemia.** Hyperlipidemia increased the levels of lipids like oxLDL in circulation, and was uptake by antigen presenting cells. T cell priming cytokines were produced, and Th17 cells secreted IL-17A, acting on M2-like tumor associated macrophages (TAM). These results maintained tumor cell proliferation, stemness, angiogenesis and tumor development, in which aspects could be inhibited by Emodin.

Overall, these results indicate that Emodin forms stable, thermodynamically favorable complexes with IL17RA and TNFR1 in the tumor microenvironment to exerts anti-tumorigenic effects. The schematic diagram of this process was depicted in Fig. 10.

## 4 Discussion

This study tried to explore the putative interaction between breast cancer development and the hyperlipidemia microenvironment, and checked the effects of natural compound Emodin in reducing tumorigenesis. Our data suggested HFD increased breast cancer development in WT mice, and Emodin suppressed tumorigenesis in the HFD-feeding mice. The mechanism was related to suppressing pro-tumorigenic macrophages and inhibiting IL-17 signaling activation.

Our *in vitro* findings provide mechanistic insights into how Emodin counteracts the tumor-promoting effects of hyperlipidemia in breast cancer. Elevated levels of oxidized low-density lipoprotein (oxLDL), a hallmark of hyperlipidemia, have been implicated in tumor progression through immune modulation [11]. These activated DC subsequently primed Th17 differentiation and IL-17 production, consistent with the established role of oxLDL in promoting IL-6/STAT3 and TGF-β-dependent Th17 polarization [12,13]. The elevated IL-17 levels have pro-tumorigenic functions, including enhanced cancer cell proliferation, migration, EMT induction, stemness and angiogenesis [14,15].

The anti-tumor efficacy of Emodin in our experiments can be attributed to its multifaceted actions: (1) Inhibition of oxLDL-induced DC activation: In line with previous studies, we observed that oxLDL robustly activated DC, as evidenced by increased expression of inflammatory cytokines TNF-α and IL-1β, followed by inhibition of Emodin. (2) Suppression of IL17-enhanced cancer cell proliferation and migration: Emodin treatment significantly induced cell apoptosis indicated by increased caspase-9 and bax, and attenuated IL17-enhanced cancer cell migration indicated by wound-healing assay. (3) Decreased tumorigenic stemness: Emodin decreased the cell population of CD24^−^CD44^+^ stem cells in E0771, both in the situation without or with IL-17A treatment. (4) Inhibited tumor growth: *in vivo* experiments showed HFD increased breast cancer development, and was suppressed by Emodin treatment. (5) Modulation of immune microcirculation in tumor: Emodin suppressed pro-tumorigenic macrophage population. (6) Suppression of angiogenesis in tumor: Emodin suppressed VEGFA indicated angiogenesis and IL-17RA expression in tumor. (7) Targets for Emodin in the anti-tumor effects: molecular docking and molecular dynamic analysis suggested Emodin-IL17RA and Emodin-TNFR1 were binding complexes during their effects.

Our data suggest that Emodin could effect on the oxLDL-APC-IL17 axis, as well as suppressing pro-tumorigenic macrophages, and these effects may synergize with its direct anti-cancer properties. This mechanism positions Emodin as a promising candidate for targeting hyperlipidemia-associated breast cancer. Further studies are warranted to validate these findings and explore potential mechanisms for these effects.

## 5 Conclusion

This study showed effectiveness of the natural compound Emodin in reducing tumorigenesis in HFD-feeding WT mice, accompanied with inhibited IL-17 activation and suppressed macrophages. This pilot study result provided evidence for the pro-tumorigenic role of hyperlipidemia in breast cancer development, and support the natural compound Emodin as a promising anti-tumor agent with targeting IL-17 signaling molecules.

## Authors’ contributions

QL designed the study and finalized the manuscript. QQL conducted experiments and LJZ completed bioinformatics analysis. YD made contributions to supervise the manuscript revision. All authors read, revised and approved the final manuscript.

## Funding

This study was supported by Municipal School (College) Joint Funding Project of 10.13039/501100020084Guangzhou Science and Technology Bureau (No. 2060206, to Q.L.), 10.13039/501100001809National Natural Science Foundation of China (No. 82274279, to Q.L.), State Key Laboratory of Traditional Chinese Medicine Syndrome Project (No. QZ2023ZZ38, to Q.L.) and Guangzhou University of Traditional Chinese Medicine-Young Top notch Talents Unveiling and Leading Project (2026, to Q.L.).

## Declaration of competing interest

There are no financial, personal interests or beliefs that may affect their objectivity.

## Data Availability

The data that has been used is confidential.
