# Supplementary material for: Emodin inhibits breast tumorigenesis in the comorbidity of hyperlipidemia and associated with IL-17 suppression
Source: Biochem Biophys Rep. 2026 Feb 26;45:102520. doi: 10.1016/j.bbrep.2026.102520 (PMC12963909; doi:10.1016/j.bbrep.2026.102520)
Supplement: Multimedia component 1 [file mmc1.docx]

1. This study adhered to the ARRIVE guidelines to ensure the transparency and scientific validity of the animal experiment. The experimental protocol was approved by the Ethics Committee of the Laboratory Animal Center, Guangdong Hospital of Traditional Chinese Medicine / Guangdong Academy of Chinese Medical Sciences (approval no.: 2024050), guaranteeing animal welfare and ethical compliance. We selected female mice as the experimental animals since breast cancer is more prevalent in females. Using female mice helps accurately simulate the development of human breast cancer and yields more clinically relevant results. During the study, we considered the potential impact of gender on the results and performed proper statistical analysis to ensure the reliability and validity of the findings.

2. Figure1a is a graph from the database, no ruler provided.
